# Treatment of infants with Syndromic Robin sequence with modified palatal plates: a minimally invasive treatment option

**DOI:** 10.1186/s13005-017-0137-1

**Published:** 2017-03-30

**Authors:** Silvia Müller-Hagedorn, Wolfgang Buchenau, Jörg Arand, Margit Bacher, Christian F. Poets

**Affiliations:** 1 Interdisciplinary Centre for Craniofacial Malformations, Tuebingen University Hospital, Calwerstrasse 7, 72076 Tuebingen, Germany; 2 Department of Neonatology, Tuebingen University Hospital, Calwerstrasse 7, 72076 Tuebingen, Germany; 3 Department of Orthodontics, Tuebingen University Hospital, Osianderstrasse 2-8, 72076 Tuebingen, Germany; 4 BIP - Orthodontic Practice, Schweickhardtstrasse 11, 72072 Tübingen, Germany

**Keywords:** Upper airway obstruction, Syndromic Robin sequence, Palatal plate, Orthodontic treatment

## Abstract

**Background:**

Infants with Robin sequence (RS) suffer from upper airway obstruction (UAO) and feeding problems. We developed an oral appliance with a velar extension in combination with functional treatment and appropriate feeding techniques, which was proven effective in isolated RS. As the above problems are particularly challenging in syndromic RS, we set out to evaluate our treatment concept also in these patients.

**Methods:**

We searched our electronic departmental database to identify all children admitted to our department between 01/01/2003 and 31/12/2009 because of syndromic RS. UAO was quantified by cardiorespiratory sleep studies performed before and during treatment with a modified palatal plate. This appliance consists of a palatal part, covering the hard palate as well as the alveolar ridges and the potential cleft, and a velar extension shifting the tongue in a more anterior position, thereby opening the pharyngeal airway. It is adjusted by fiberoptic nasopharyngoscopy and controlled by cardiorespiratory sleep studies. Obstructive sleep apnea was defined as a mixed obstructive sleep apnea index (MOAI) >3/h. Feeding modalities before and after treatment and weight gain, determined as standard deviation score, were also evaluated.

**Results:**

Of 68 children meeting inclusion criteria, 56 completed treatment (46 of these being infants). Underlying diagnoses included craniofacial dysostosis (*N* = 13) and synostosis syndromes (*N* = 5), unspecified dysmorphic syndromes (*N* = 23) and miscellaneous rare conditions (*N* = 27).

Median MOAI decreased from 8.5 (range 0.3–76.0) at admission to 1.1 (0.0–5.2) at discharge (p < 0.001). 51 children received only a TPP and 5 additionally continuous positive airway pressure (CPAP) or high-flow nasal cannula during sleep for mild residual OSA. Three children ultimately required tracheostomy. The number of exclusively gavage fed infants was reduced from 23 to 7. Conversely, the number of children fed exclusively by mouth increased from 18 to 30. Median SDS for weight decreased from −1.6 (−3.5–1.7) to −1.3 (−4.1-2.5). Twelve children had their treatment prematurely discontinued, e.g. due to laryngeal collapse/laryngomalacia. No patient died during treatment.

**Conclusion:**

Treatment of UAO and feeding problems in these children with syndromic RS by a modified palatal plate with a velar extension was shown to be effective and safe. If confirmed in prospective studies, it may help to avoid more invasive interventions.

## Background

Robin Sequence (RS) is characterized by micrognathia, glossoptosis and respiratory distress; a U-shaped cleft palate may also occur [1]. Its incidence ranges from 1:8500 to 1:14500 live births [2, 3]. An epidemiological study determined a birth prevalence of 12.4 per 100.000 live births in Germany [4]. Recently, recommendations for the initial evaluation of RS and clinical descriptors were published in a consensus report [5].

The main clinical problems in RS include upper airway obstruction (UAO), feeding difficulties and failure to thrive, potentially leading to neurocognitive impairment, behavioral difficulties, pulmonary hypertension, congestive heart failure and sudden death [6]. Feeding problems and failure to thrive may be secondary to respiratory problems, i.e. an increased work of breathing [7], but may also be due to swallowing dysfunction [8] and abnormal sucking quality [9], leading to a low calory intake, pulmonary aspiration, and gastro-esophageal reflux. Interventions for children with RS should thus be assessed by their ability to improve respiratory problems and weight gain.

RS may occur both, as an isolated entity (isolated RS) or as a component of a syndrome, i.e. in association with other malformations (syndromic RS). More than 50 syndromes have been described in association with RS [10], the most common being Stickler syndrome, Nager syndrome, Treacher Collins syndrome and 22q11 deletion (velocardiofacial syndrome) [11]. In a recent study, 40% of RS cases were isolated and 60% syndromic [12]. Thus, failure to thrive in RS may also be caused by an underlying syndromic condition [13].

Endoscopically, 4 types of UAO can be differentiated [14], of which a so-called type 1 UAO (i.e., true glossoptosis) is found in 90% of patients with isolated RS, but in as little as 44% of those with syndromic RS [15]. Treatment of UAO in RS should stabilize the pharyngeal wall and widen the hypopharynx by shifting the tongue into a more anterior position, thereby facilitating oral feeding and stimulating mandibular growth. Most current treatment options, however, fail these aims or are considerably invasive [16].

**Fig. 1:**
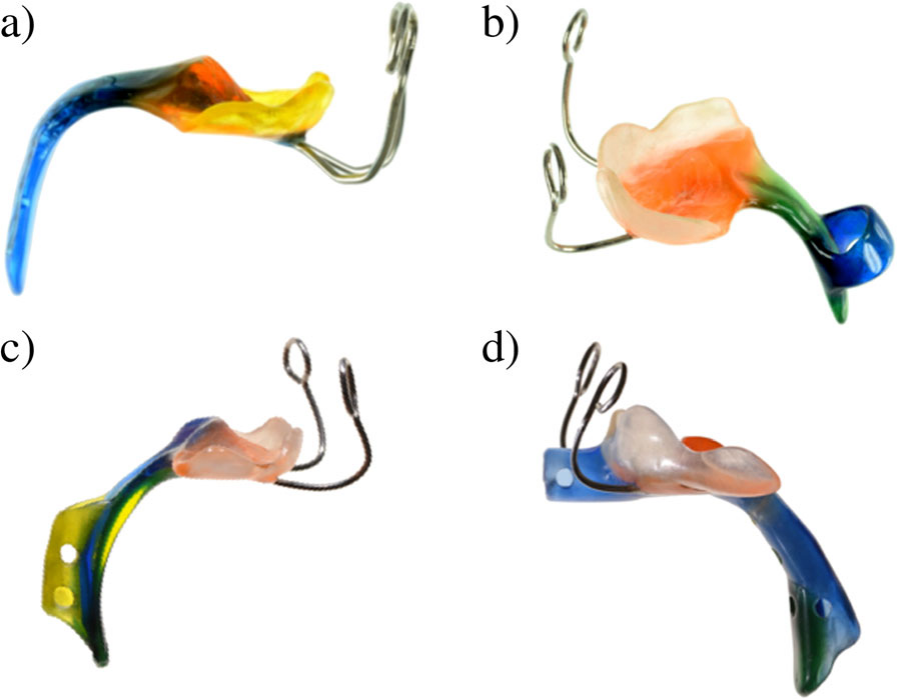
Various types of palatal plates used in these patients: **a** classical Tübingen palatal plate; **b** modified plate with a ring or **c** a tube attached to the extension; **d** modified plate with a tube that has an extra-oral extension

In our centre, an oral appliance (Tübingen Palatal Plate (TPP) or pre-epiglottic baton plate) (Fig. 1a) in combination with Manual Orofacial Therapy (MOT) according to Castillo-Morales [17] and appropriate feeding techniques are used as a first line treatment option for infants with RS and UAO. The TPP consists of a plate with a velar extension that shifts the tongue forward, thereby widening the hypopharynx and releasing the UAO. This treatment concept has been successfully evaluated in infants with isolated RS [18, 19], but little is known whether it is equally effective in syndromic RS.

The aim of this study was to evaluate our treatment approach in the latter patient group, again focusing on UAO and feeding problems. Because of their more complex anatomical and functional disturbances, modifications to the original TPP were often necessary, which will also be described here.

## Methods

### Patients

The department’s electronic patient database (Neodat®, Tübingen, Germany) was searched for all children with a diagnosis of syndromic RS admitted to the Department of Neonatology at Tübingen University Hospital between 01/01/2003 and 31/12/2009. Children with isolated RS or Down syndrome were excluded. In total, of 68 children (29 females) meeting inclusion criteria, 12 had treatment discontinued for various reasons (see below), so that data were analyzed for 56 children completing treatment.

### Study design and procedures

All patients underwent orthodontic treatment by TPP in combination with MOT and an intensive feeding training. They also had sleep studies performed upon admission, after fitting the TPP and after each modification of the plate. The following clinical data were collected: underlying diagnoses, age, gender, body weight, length, head circumference, type of cleft, treatment prior to admission, feeding modalities before and after treatment with the TPP as well as type of palatal plate used and sleep study results.

### Sleep studies

Sleep studies were performed using a computerized system (Embla N 7000, MedCare, Reykjavik, Iceland). The study montage comprised the following channels and sensors: chest and abdominal wall movements (respiratory inductive plethysmography, MedCare), nasal pressure and linearized nasal airflow (nasal prongs and built-in pressure transducer, MedCare), pulse oximeter saturation (SpO_2_) and pulse waveform (Radical, Masimo Inc., Irvine, USA), electrocardiography (ECG, MedCare), and digital video via infrared camera (Panasonic; Tokyo, Japan). Recordings commenced in the evening and lasted for at least 8 h; all infants were studied in the supine position.

All recordings were manually analyzed by a trained scorer not involved in clinical management and blinded to their timing (before/after treatment) for the presence of respiratory events using standard criteria [20]. In brief, total sleep time (TST) was determined from the first 10-min epoch without movement artifact or a distorted pulse waveform to the last such 10-min epoch; recordings comprising less than 3 h of TST were excluded. An apnea was scored if (i) the amplitude of the nasal airflow fell to <20% of the average amplitude of the two preceding breaths, (ii) no airflow was detected at the mouth, and (iii) the event comprised at least two breath cycles (i.e. approximately 3–4 s). An obstructive apnea (OA) was scored if (i) the above criteria for apnea were fulfilled and (ii) out-of-phase movements of the chest and abdomen were present. A central apnea was scored if (i) criteria for apnea were fulfilled and (ii) no chest and abdominal wall movements were present. Mixed apneas were defined as those with both a central and obstructive component, each lasting at least two breath cycles. A mixed obstructive apnea index (MOAI) was calculated as the sum of mixed apneas plus OA per hour of TST. To comply with our previous studies on this patient group, obstructive sleep apnea (OSA) was defined as a mixed-obstructive apnea index (MOAI) >3. Infant polysomnography has a high validity and reproducibility [21].

Desaturation events were visually confirmed to exclude spuriously low values. Events with a distorted pulse waveform signal within 7 s prior to their onset were considered artifactual and excluded. The number of desaturation events to ≤80% SpO_2_ was counted and expressed as indices, defined as events per hour of TST (DI_80_).

### Treatment protocol

After diagnosis, children were admitted and monitored in the neonatal intermediate care unit where they also underwent a baseline cardiorespiratory sleep study and fiberoptic nasopharyngoscopy without sedation to assess the type and localization of the UAO. This endoscopy usually took only 4–5 min, although we never measured this systematically.

**Fig. 2:**
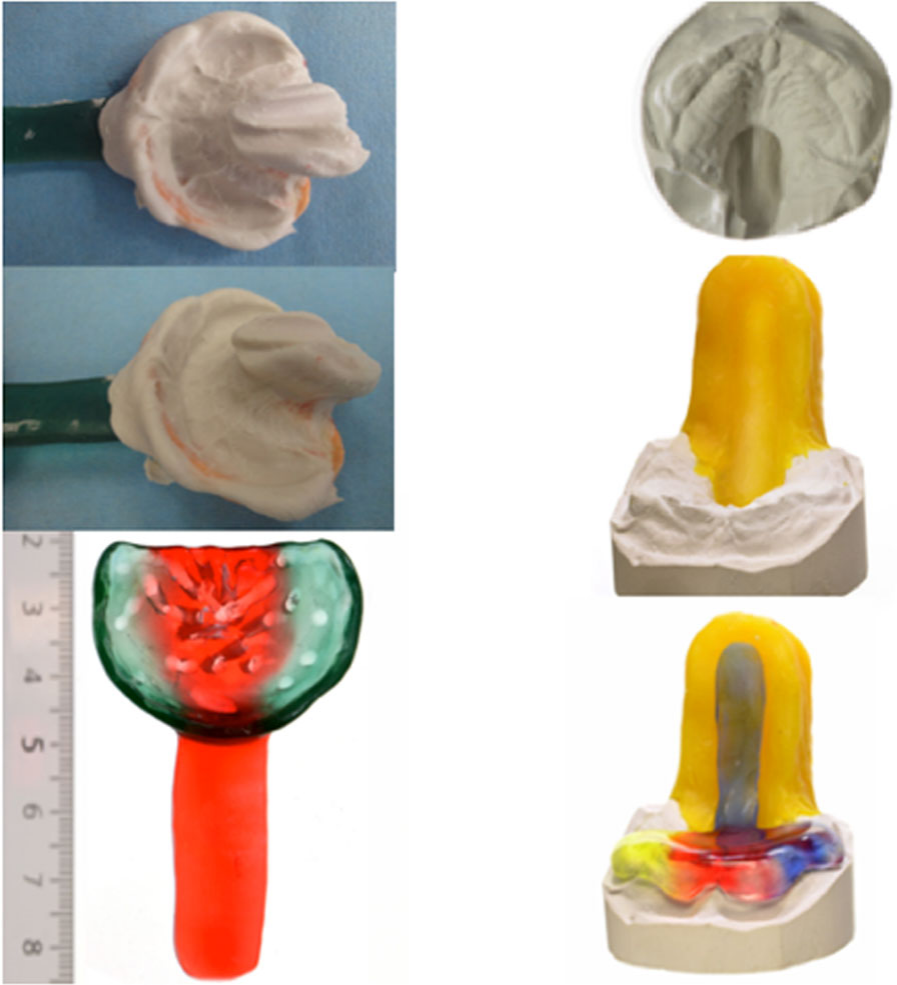
Fabrication of a prototype of the plate: *left*, impression tray and imprint, *right*, plaster cast and molding of the velar extension, resulting in the prototype (*bottom left*)

Next, infants had a maxillary imprint taken using a custom-made impression tray and alginate (Tetrachrom-Super-Alginat, ISO 1563, Klasse B, Typ I, Kaniedenta, Herford, Germany). This imprint covered the entire hard palate, the alveolar ridges and the vestibule. This procedure was carried out in the neonatal intermediate care unit under cardiorespiratory monitoring without sedation, but with a nasopharyngeal airway in place and in the presence of an experienced neonatologist. Then a plaster cast was produced using high precision dental plaster (Girodur Type IV, Synthetic Superhard Stone Plaster for Sectioned and Master Models DIN EN 26873, white, Girrbach Dental GmbH, Pforzheim, Germany). Using this cast, appliances were made from hard acrylic (autopolymerizing methylmethacrylate, Orthocryl, Dentaurum, Pforzheim, Germany). The TPP consisted of a palatal part that covered the hard palate and the cleft as well as the alveolar ridges and a velar extension of approximately 3 cm in length. The shape of the velar extension was modeled from dental wax and attached dorsally to the plaster cast. The length and the angle of the extension were chosen so that it was adjacent to the dorsum of the tongue. It was manufactured using a blue color to facilitate endoscopic evaluation in situ. After polymerization this prototype was polished using standard techniques (Fig. 2).

**Fig. 3:**
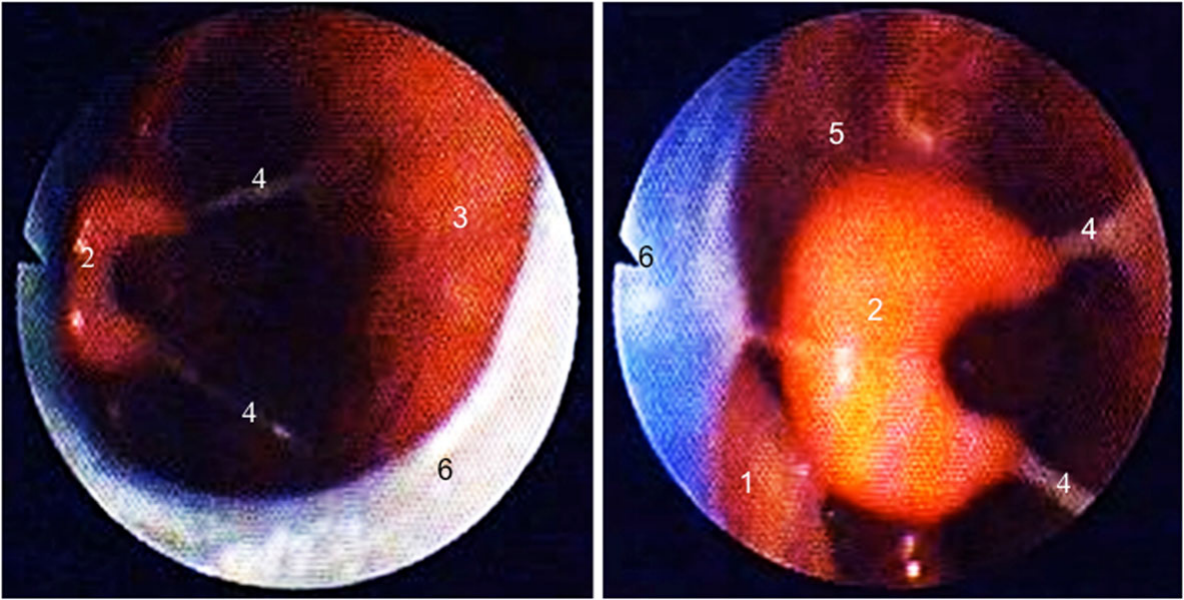
Endoscopic views of the plate in situ: *right*, a classical TPP, *left*, a TPP with a tube attached to the extension (to prevent the laryngeal structures from collapsing). 1) tongue, 2) epiglottis, 3) posterior pharyngeal wall, 4) aryepiglottic folds, 5) vallecula, 6) spur/tube of the TPP

**Fig. 4:**
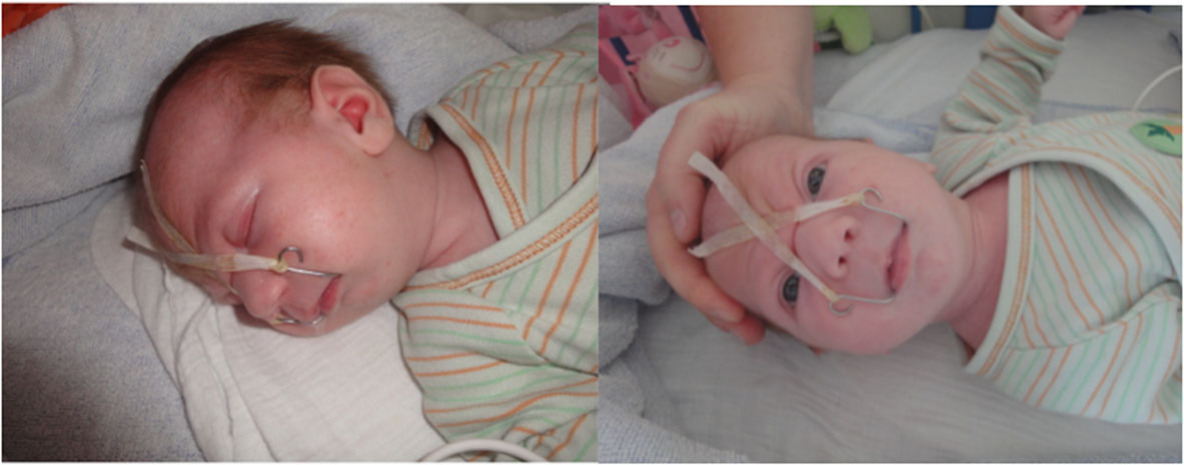
Orthodontic appliance (TPP) in situ with extraoral wires to secure optimal position. (Photographs shown with parental permission)

Once a prototype of the plate was ready, infants had a repeat endoscopy to adjust the length and angle of the velar extension. The tip of the extension descended down to the vallecula epiglottica and the angulation was responsible for the anterior shifting of the base of the tongue, erecting the epiglottis thereby widening the airway. If the airway appeared endoscopically (Fig. 3) and clinically open, the prototype was finished, a strengthening wire incorporated into the extension to safeguard the device against mechanical failure and extraoral wires were added to improve retention of the plate (Fig. 4). Two days later, its effectiveness was assessed by a second sleep study. If this sleep study still showed a MOAI >3, the plate was modified: if compression of the soft palate against the pharyngeal wall by the tongue or an inward movement or collapse of the pharyngeal walls and circular constriction of the pharynx was seen, a ring or a perforated tube with or without an extraoral extension were attached to the velar extension (Fig. 1 b-d). Again, effectiveness of the modified plate was controlled endoscopically followed by a sleep study. Treatment also comprised appropriate feeding techniques (finger feeding and Playtex Drop-Ins®, Playtex Products) and an orofacial stimulation therapy according to Castillo Morales. Usually, plates are also worn during feeds.

In infants and during the first 2–3 months of treatment, appliances are recommended to be worn for 24 h a day and are removed only 1x/day for cleaning purposes. Depending on the (subjective) degree of mandibular catch-up growth, they are then only worn at nighttime. Fitting the plate usually takes less than 10s. The appliances are held in situ by adhesion and suction and by an adhesive cream (Blend-a-dent Super Haftcreme, Procter & Gamble, Schwalbach, Germany) to improve retention. The extraoral bows are fixed to the face with adhesives (Steri-Strip, 3 M Helath Care, St. Paul, MN). Fitting of the plate is controlled at each outpatient visit. If the palatal part becomes too small a new TPP has to be adapted. After discharge, the patients are seen at intervals of 6 to 8 weeks at the orthodontic outpatient clinic; a further sleep study is mandatory 3 months after discharge. In general, a new plate is necessary after 3 months in infancy or if a notch appears on the alveolar ridges, due to a then too small palatal part of the TPP or if sleep study results deteriorate.

### Statistical analysis

Results are reported as median and range. Comparisons between sleep study results were done using software (Statistical Package for the Social Science, Version 18, IBM, New York, USA). For the analysis of sleep parameters, the Wilcoxon signed rank test was used.

## Results

**Fig. 5:**
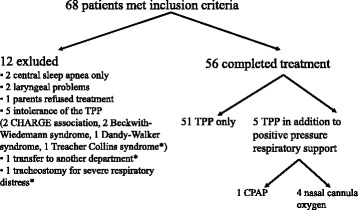
Patient flow (* Asterix indicates children who underwent tracheostomy)

Out of 68 children meeting inclusion criteria, 12 had TPP treatment discontinued prematurely (Fig. 5): in 4 cases TPP proved not to be indicated (two had no upper airway obstruction but only central sleep apnea, the other 2 had laryngeal collapse/laryngomalacia). In one case parents were non-compliant and ultimately refused TPP treatment, and 5 children did not tolerate the plate (2 were diagnosed with CHARGE association, and one each with Dandy-Walker-syndrome, Beckwith-Wiedemann syndrome and a severe phenotype of Treacher Collins syndrome subsequently requiring tracheostomy. One child was transferred to the local department of cardiology and also underwent tracheostomy there. A third child underwent tracheostomy soon after admission because of recurrent severe respiratory distress unresponsive to palatal plate therapy. Thus, 3 out of 68 patients (4%) required tracheostomy; no patient died.

Of the remaining 56 children (35 males), 46 were infants, 31 of them being referred within the first 3 months after birth, while the remaining were between 2 and 12 years old at admission.

Underlying diagnoses (Table 1) included craniofacial dysostoses (*N* = 13) and synostosis syndromes (*N* = 5) but many patients (*N* = 23) had an unspecified dysmorphic syndrome or were suffering from one of a variety of extremely rare conditions (*N*=27) (Tables 1 and 2).

**Table 1:** Diagnoses in the patient group

Diagnoses	N	N
Craniofacial synostosis syndromes	5	
Apert syndrome		1
Crouzon syndrome		3
Pfeiffer syndrome		1
Craniofacial dysostosis syndromes	13	
Treacher Collins syndrome		9
Goldenhar syndrome		3
Nager acrofacial dystosis		1
Unspecified dysmorphic syndromes	23	
Other rare conditions	27

**Table 2:** Other rare conditions in the patient group

Rare conditions	N	Rare conditions	N
Del(4q) syndrome	1	CHARGE association	4
Cat-eye syndrome	1	Popliteal pterygium syndrome	1
Cri-du-chat syndrome	1	Robinow syndrome	1
Dandy-Walker syndrome	2	Silver Russel syndrome	1
Klinefelter syndrome	1	Single incisor syndrome	2
Loeys-Dietz syndrome	1	Cerebro-costo-mandibular syndrome	2
Miller syndrome	1	Sotos syndrome	1
Möbius sequence	1	Translocation trisomy 18	1
Mowat-Wilson syndrome	1	Trisomy 9	1
Cornelia-de-Lange syndrome	1	Partial trisomy 18 with monosomy 9	1
Beckwith-Wiedemann syndrome	1		

Concerning cleft palate, 30 children did not have any cleft, whereas 16 had a cleft of the hard and soft palate, 6 presented only a cleft of the soft palate, and 3 had a submucosal cleft. In one case cleft repair had been done elsewhere prior to referral.

Upon admission to our department, most children had already received treatment for UAO, e.g. a nasopharyngeal tube or endotracheal intubation, glossopexy, tracheostomy, nasal positive pressure ventilation or nasal cannula oxygen, conventional palatal plates or adenotomy (Table 3). Five patients, in addition to nasopharyngeal intubation, temporarily needed continuous positive airway pressure (CPAP) or high-flow nasal cannula (HFNC) support, and one child with glossopexy had initially received a nasopharyngeal airway. In 3 children a transitory switch from nasal cannula oxygen to CPAP was necessary. However, in 25 patients (mostly <3 months of age), no specific treatment for UAO had been initiated prior to admission.

**Table 3:** Pre-treatment upon admission in children completing orthodontic treatment (N = 56)

Tratment upon referral	N
None	25
Nasopharyngeal intubation	6
Endotracheal intubation	2
Glossopexy	1
Nassal postive pressure support	9
Tracheostomy	3
Other (adenotomy, conventional palatal palatal plate, nasal cannula oxygen)	10

To treat UAO, 4 types of plates were constructed depending on the type of obstruction. The classical TPP (Fig. 1a) turned out to be sufficient in 33 patients. Twenty-three children needed a modified plate to resolve UAO: 3 required a plate with a ring (Fig. 1b) and 20 with a tube of different length attached to the velar extension, i.e. an oropharyngeal airway plate (Fig. 1c, d).

At discharge, 51 out of the 56 children who completed treatment received only a (modified) TPP. Of the remaining patients, 1 received support via CPAP and 4 HFNC at night in addition to TPP treatment, because they still had had a MOAI >3. The 3 children who already had a tracheostomy in place at referral all had their tracheostomy closed prior to hospital discharge.

### Sleep study results

At baseline, all but 2 patients presented with obstructive sleep apnea, while 2 had central sleep apnea only. During treatment, sleep study results improved significantly in all parameters analyzed (Table 4).

**Table 4:** Sleep study results (Wilcoxon rank test), shown as median (range)

Parameter	Baseline sleep study	Sleep study result at discharge	*p*-value
MOAI	8.5 (0.3/76.0)	1.1 (0.0/5.2)	p < 0.001
DI80	2.3 (0.0/33.0)	0.0 (0/5.1)	p < 0.001

### Feeding, growth and weight gain

Upon admission, 23 children were fed exclusively by gavage; this number was reduced to 7 at discharge. The number of children who were fully orally fed increased from 18 to 30 during that time.

Weight, head circumference and length were determined for all infants throughout their first year of life (*n* = 46), i.e. at birth (missing values for three patients), at admission and at hospital discharge and expressed as standard deviation scores (SDS). Median SDS scores had been below 0 for all 3 parameters at birth, and had deteriorated further until admission to our department. During TPP treatment (from admission to discharge), all 3 parameters improved, although median SDS continued to be <0 and differences were not significant (Table 5).

**Table 5:** Development of weight, growth and head circumference under treatment

		Birth *n* = 43	Admittance *n* = 46	Discharge *n* = 46
Weight	SDS-value	–1.1 (–4.7/2.4)	–1.6 (–3.5/1.7)	–1.3 (–4.1/2.5)
Length	SDS-value	–0.9 (–4.9/2.3)	–1.3 (5.4/3/2)	–1.2 (–5.9/1.3)
Head circumference	SDS-value	–0.7 (–4.9/4.1)	–1.3 (–6.1/2.3)	–0.9 (–5.9/1.3)

Results are shown as median (range) SDS (standard deviation score) for patients <1 year of age only. In 3 infants, no birth weight was available from the hospital files

### Duration of hospital stay

Fourteen (25%) children stayed in hospital for less than 2 weeks, 20 (36%) for 2–4 weeks, and 22 (39%) could only be discharged home after more than 1 month of hospital stay.

## Discussion

Upper airway obstruction and feeding problems are major clinical problems in isolated RS and are even more pronounced in syndromic RS. The latter patients are more susceptible to severe and life threatening respiratory complications than their non-syndromic counterparts [13] and have poorer outcomes concerning growth and development in spite of early intervention and good control of airway and feeding problems compared to children with isolated RS [22, 23].

It has been estimated that up to 80% of RS are associated with another syndrome [24]. In syndromic RS different pathophysiologic mechanisms may be involved, with all 4 types of obstruction described by Sher [14] potentially contributing to the UAO, as also confirmed in our cohort. In our experience, the type of obstruction determines the respective modification of the plate. If a type 2 obstruction is observed endoscopically, a plate with a tubular structure attached to the velar extension may be necessary to bridge the obstruction, due to the tongue displacing the velum posteriorly against the pharyngeal wall. If the obstruction is due to an inward movement of the lateral pharyngeal wall or pharyngeal constriction (Sher types 3&4), the velar extension requires attachment of either such a tube or a ring to open the supra-laryngeal space (Fig. 1b-d).

No child was diagnosed with Stickler syndrome, even if this is the most frequent syndrome described in association with RS. Stickler syndrome was likely underdiagnosed in our rather young cohort, even though all patients were seen by a geneticist and an ophthalmologist during their hospital stay, as no routine genetic testing was done for this condition.

We were able to treat UAO successfully in 51 of the 68 cases referred (75%) with syndromic RS, and 80% of those considered candidates for TPP treatment (i.e., after exclusion of those with central sleep apnea and laryngeal problems), only by using the TPP and its modifications. Five additional children were treated by TPP in association with CPAP or high-flow nasal cannula because they continued to suffer from a mild degree of OSA (MOAI > 3) and we could not bring the velar extension into a steeper position. Furthermore, 5 children (7%) ultimately did not tolerate the TPP, which became apparent after 2–3 weeks of treatment. Only 3 children (4%) underwent tracheostomy.

Other treatment protocols proposed for RS, such as pneumatic airway stenting with nasal CPAP or use of a nasopharyngeal airway, provide no growth stimulus for the mandible. This is why we added rather than replaced CPAP or high-flow nasal cannula to TPP treatment in case of persisting UAO during TPP treatment. Other groups reported CPAP as the preferred treatment for UAO in RS [25], but acquired maxillary hypoplasia secondary to the prolonged use of CPAP has been reported [26].

In infants with isolated RS, effectiveness of the TPP in improving UAO may be explained by its velar extension shifting the base of the tongue forward, thereby widening the pharyngeal airway (immediate effect) [18, 19]. The second aim of the TPP and its modifications is to push the tongue forward and we assume that this protrusion may induce a mandibular growth stimulus [27] (long-term effect), although his has yet to be proven by systematic measurements currently underway in our center.

Main reasons for non-tolerance of the plate were repetitive impression marks and occasionally retching or vomiting. A severe swallowing disorder, as seen in 2 of our patients with CHARGE association, or a large tongue, as seen in two other patients with Beckwith-Wiedemann syndrome, also seemed to prohibit acceptance of the plate. In our experience a severe swallowing disorder is a contraindication to TPP treatment. In addition, the TPP proved ineffective in 2 patients presenting with laryngeal problems, which may accompany syndromic RS, as also reported by others [13].

We used the MOAI as our primary outcome parameter because, at least in infants, obstructive and mixed apneas share the same pathophysiology [28], i.e. upper airway narrowing, which is improved by the TPP. A potential limitation of our study is that we have no center or patient-group specific data on the inter-observer variability of the MOAI.

Airway obstruction may become more severe in the second month of life [29]. Therefore, special attention has to be paid also to infants with only minimal signs of UAO shortly after birth. This might be an explanation why 25 patients had no treatment prior to admission. Nonetheless, we cannot exclude a later development of UAO in some patients. Robison [30] found that 35 of 48 children with RS had OSA or sleep disordered breathing, many of which presented after their 1^st^ year of life and after cleft palate repair. Late presentations of RS have also been reported by others [31], suggesting that long-term follow-up of this patient group, including sleep studies, is important. We wish to stress that the palatal plate therapy described here is also possible beyond infancy, although in our experience, treatment will be completed much faster if started in the first 1–3 postnatal months.

In our cohort, the proportion of children treated with TPP (*n* =56) that could be fed exclusively by mouth increased from 18 (32%) at referral to 30 (54%) at discharge. We consider this encouraging result to be related to the TPP, in combination with our functional oral treatment protocol, both of which help normalizing the tongue position by shifting it into a more anterior position.

Airway management in children with isolated or syndromic RS is still a challenge: many treatment options are reported in the literature [32], with no consensus about the ideal treatment. While we clearly show that the treatment modality reported here improves airway obstruction and failure to thrive, interpretability is limited by our retrospective study design. Also, data on mandibular (catch-up) growth during TPP treatment are needed. This, however, is also true for most other modalities currently applied. While such data are urgently awaited, the effectiveness and side effects of the treatment reported here may be compared to those reported for other interventions.

## Conclusion

Treatment of UAO in children with syndromic RS with modified TPP was shown to be an effective, safe and only minimally invasive option. It helps to avoid more invasive interventions. Furthermore, we consider it a causal treatment, which may induce growth of the hypoplastic mandible and counteracts potential oropharyngeal and muscular deficiencies. It requires an interdisciplinary team consisting of neonatologists trained in nasopharyngeal endoscopy, orthodontists, pediatric sleep specialists, and speech therapists familiar with orofacial regulation therapy. The nursing team is also of outstanding importance, not least to train the parents in handling the plate. It has to be pointed out that this kind of treatment is more difficult to apply in patients with syndromic compared to isolated RS and often requires a longer hospital stay than seen in the latter group. Long-term follow-up is also necessary and currently underway in these patients.

## Abbreviations

CPAP: Continuous positive airway pressure
HFNC: High-flow nasal cannula
MOAI: Mixed obstructive apnea index
MOT: Manual orofacial therapy
OA: Obstructive apnea
OSA: Obstructive sleep apnea
RS: Robin sequence
SDS: Standard deviation score
TPP: Tuebingen palatal plate
TST: Total sleep time
UAO: Upper airway obstruction
